# The genome sequence of the Purple Clay moth, *Diarsia brunnea *(Denis & Schiffermüller) 1775

**DOI:** 10.12688/wellcomeopenres.22868.1

**Published:** 2024-08-14

**Authors:** Jo Davis, Dougie Menzies, Wellcome Sanger Institute Tree of Life Management, Samples and Laboratory team, Wellcome Sanger Institute Scientific Operations: Sequencing Operations, Wellcome Sanger Institute Tree of Life Core Informatics team, Tree of Life Core Informatics collective, Darwin Tree of Life Consortium

**Affiliations:** 1 Independent researcher, Lanark, South Lanarkshire, Scotland, UK; 2 Bute Museum & Natural History Society, Rothesay Isle of Bute, Scotland, UK

**Keywords:** Diarsia brunnea, Purple Clay moth, genome sequence, chromosomal, Lepidoptera

## Abstract

We present a genome assembly from an individual male *Diarsia brunnea* (the Purple Clay moth; Arthropoda; Insecta; Lepidoptera; Noctuidae). The genome sequence has a total length of 586.80 megabases. Most of the assembly is scaffolded into 31 chromosomal pseudomolecules, including the Z sex chromosome. The mitochondrial genome has also been assembled and is 15.29 kilobases in length. Gene annotation of this assembly on Ensembl identified 18,730 protein-coding genes.

## Species taxonomy

Eukaryota; Opisthokonta; Metazoa; Eumetazoa; Bilateria; Protostomia; Ecdysozoa; Panarthropoda; Arthropoda; Mandibulata; Pancrustacea; Hexapoda; Insecta; Dicondylia; Pterygota; Neoptera; Endopterygota; Amphiesmenoptera; Lepidoptera; Glossata; Neolepidoptera; Heteroneura; Ditrysia; Obtectomera; Noctuoidea; Noctuidae; Noctuinae; Noctuini; *Diarsia*; *Diarsia brunnea* (Denis & Schiffermüller) 1775 (NCBI:txid987923).

## Background

The Purple Clay, *Diarsia brunnea*, is a macromoth of the Noctuidae (Noctuid) family, found throughout Britain and Ireland and across Europe and Asia as far as China and Japan ( GBIF Secretariat, 2024).

In the UK its habitat is varied and includes broadleaved and mixed woodlands, moors, scrubby heathland, hedgerows and gardens. It is on the wing from late June to August. The Purple Clay is described as common, but not abundant, with its numbers probably declining though its distribution has not decreased ( Randle *et al.*, 2019)

It is an attractive moth and the dry description given in modern textbooks does not give it the justice that a late nineteenth Lepidopterist does, who writes of the Purple Clay as ‘beautiful……with its rich purple and red-brown ground colour, ochreous marblings and pale ochreous-grey reniform’ ( Tutt, 1896). The vernacular name ‘Purple’ refers to the purple tinge so evident in a fresh specimen, whereas its scientific name ‘ *brunnea*’ refers to the brown undertone.

The Purple Clay hibernates as a caterpillar. The larvae feed by night retiring to leaf litter during the day. The diet of the larvae is truly catholic with a wide range of herbaceous plants in the autumn and in the spring more woody plants and even bracken.

The specimen was collected from a mercury vapour moth trap set up overnight on 16 June 2022 in Little Sparta, a garden designed by the artist Ian Hamilton Finlay in the 1960s, in South Lanarkshire, Scotland, surrounded by moorland and sheep farms. This species was identified by Jo Davis, an amateur lepidopterist and member of Butterfly Conservation.

## Genome sequence report

**Figure 1.**
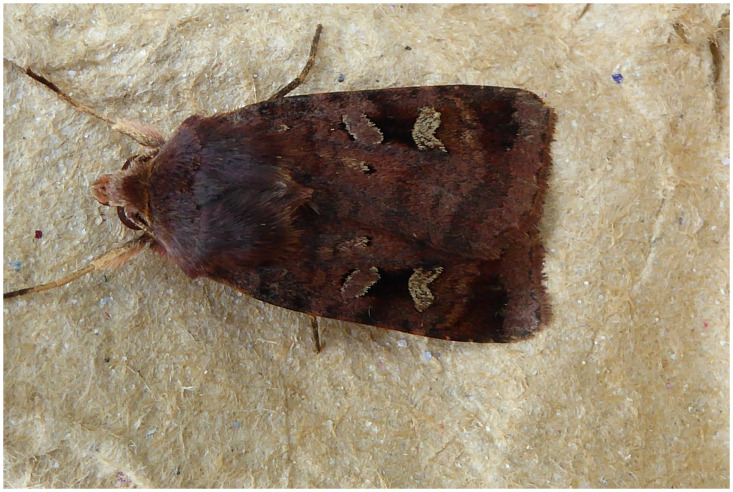
Photograph of the *Diarsia brunnea* (ilDiaBrun1) specimen used for genome sequencing.

The genome of an adult *Diarsia brunnea* ( Figure 1) was sequenced using Pacific Biosciences single-molecule HiFi long reads, generating a total of 21.90 Gb (gigabases) from 2.13 million reads, providing approximately 36-fold coverage. Primary assembly contigs were scaffolded with chromosome conformation Hi-C data, which produced 122.07 Gbp from 808.42 million reads, yielding an approximate coverage of 208-fold. Specimen and sequencing information is summarised in Table 1.

**Table 1.** Specimen and sequencing data for *Diarsia brunnea*.

Project information			
**Study title**	Diarsia brunnea (purple clay)		
**Umbrella BioProject**	PRJEB60714		
**Species**	*Diarsia brunnea*		
**BioSample**	SAMEA112198376		
**NCBI taxonomy ID**	987923		
Specimen information			
**Technology**	**ToLID**	**BioSample** **accession**	**Organism part**
**PacBio long read sequencing**	ilDiaBrun1	SAMEA112198436	thorax
**Hi-C sequencing**	ilDiaBrun2	SAMEA112198555	head
**RNA sequencing**	ilDiaBrun2	SAMEA112198557	abdomen
Sequencing information			
**Platform**	**Run accession**	**Read count**	**Base count (Gb)**
**Hi-C Illumina NovaSeq 6000**	ERR11040192	8.08e+08	122.07
**PacBio Sequel IIe**	ERR11029701	2.13e+06	21.9
**RNA Illumina NovaSeq X**	ERR12765140	5.89e+07	8.89

**Figure 2.**
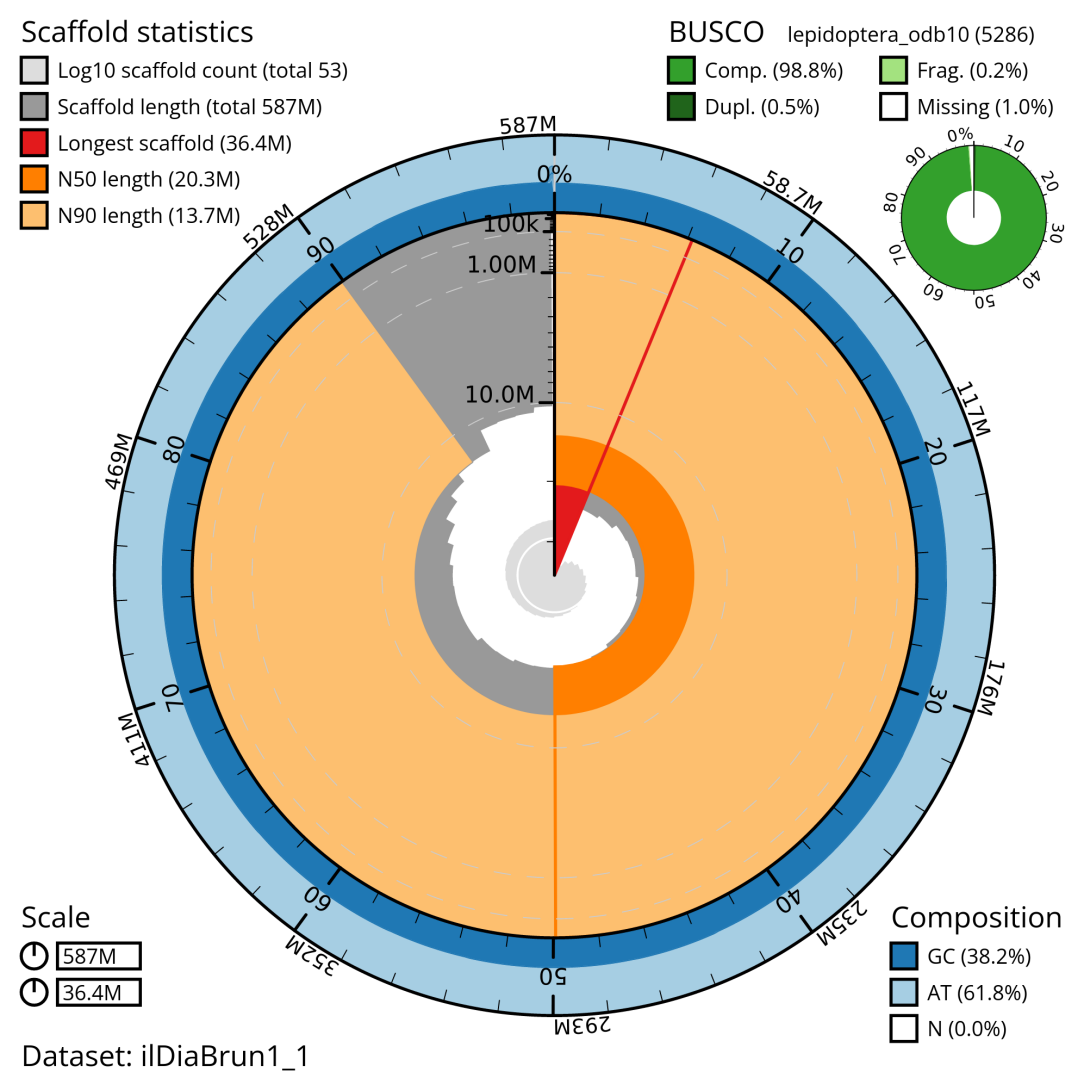
Genome assembly of *Diarsia brunnea*, ilDiaBrun1.1: metrics. The BlobToolKit snail plot shows N50 metrics and BUSCO gene completeness. The main plot is divided into 1,000 size-ordered bins around the circumference with each bin representing 0.1% of the 586,790,279 bp assembly. The distribution of scaffold lengths is shown in dark grey with the plot radius scaled to the longest scaffold present in the assembly (36,429,188 bp, shown in red). Orange and pale-orange arcs show the N50 and N90 scaffold lengths (20,336,188 and 13,737,000 bp), respectively. The pale grey spiral shows the cumulative scaffold count on a log scale with white scale lines showing successive orders of magnitude. The blue and pale-blue area around the outside of the plot shows the distribution of GC, AT and N percentages in the same bins as the inner plot. A summary of complete, fragmented, duplicated and missing BUSCO genes in the lepidoptera_odb10 set is shown in the top right. An interactive version of this figure is available at https://blobtoolkit.genomehubs.org/view/ilDiaBrun1_1/dataset/ilDiaBrun1_1/snail.

**Figure 3.**
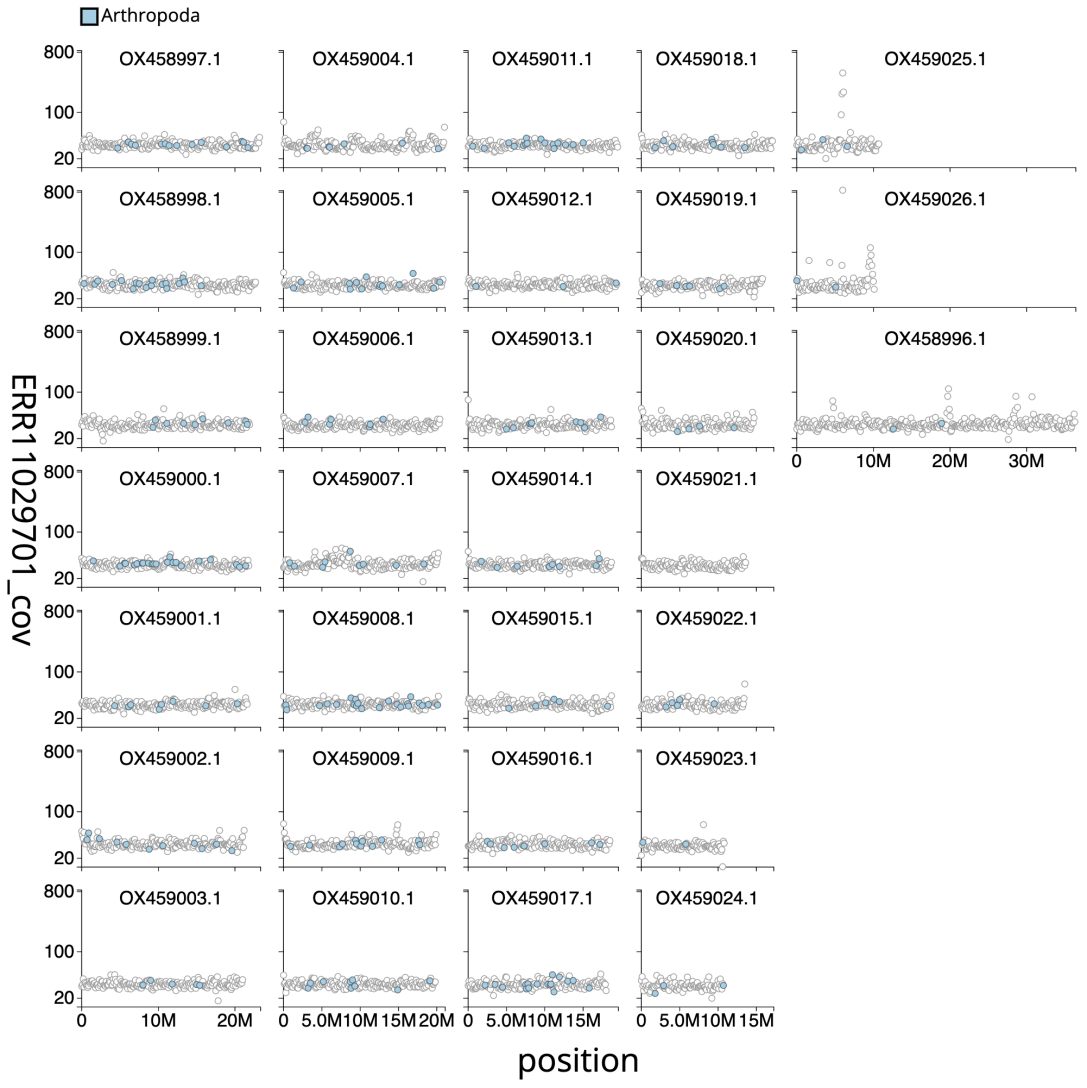
Genome assembly of *Diarsia brunnea*, ilDiaBrun1.1: Distribution plot of base coverage in ERR11029701 against position for sequences in assembly ilDiaBrun1_1. Windows of 100kb are coloured by phylum. The assembly has been filtered to exclude sequences with length < 2,550,000. An interactive version of this figure is available here.

**Figure 4.**
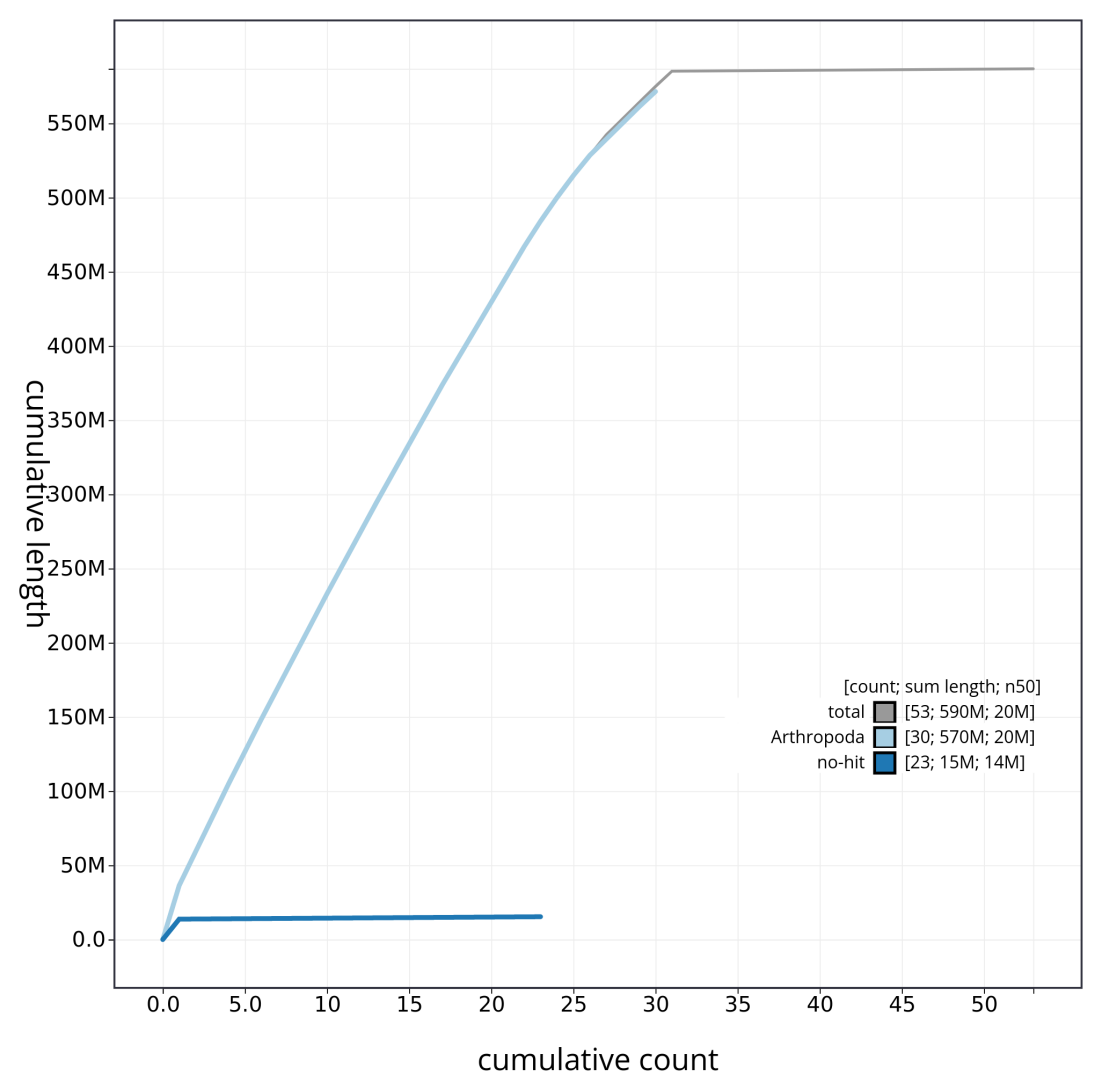
Genome assembly of *Diarsia brunnea* ilDiaBrun1.1: BlobToolKit cumulative sequence plot. The grey line shows cumulative length for all sequences. Coloured lines show cumulative lengths of sequences assigned to each phylum using the buscogenes taxrule. An interactive version of this figure is available at https://blobtoolkit.genomehubs.org/view/ilDiaBrun1_1/dataset/ilDiaBrun1_1/cumulative.

**Figure 5.**
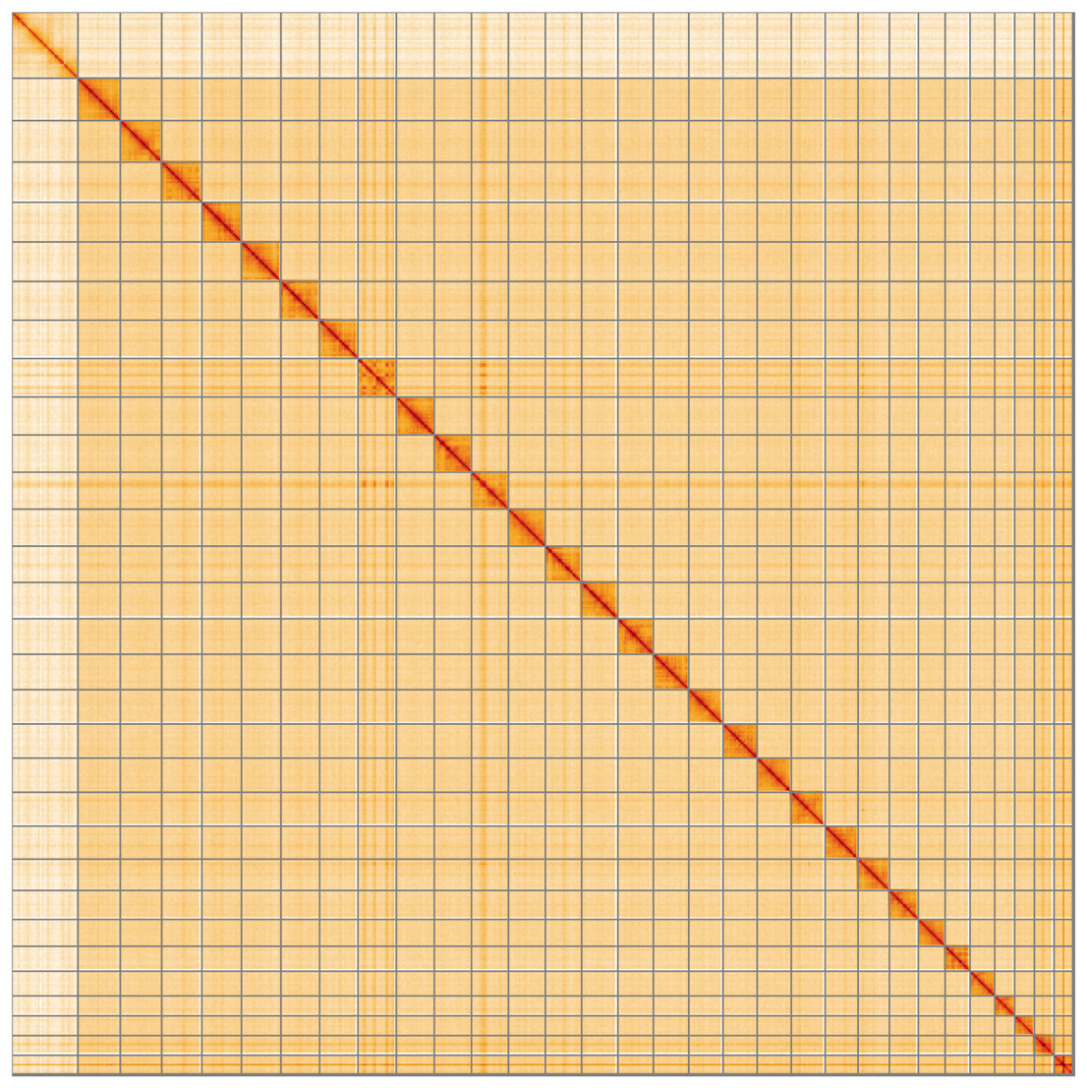
Genome assembly of *Diarsia brunnea* ilDiaBrun1.1: Hi-C contact map of the ilDiaBrun1.1 assembly, visualised using HiGlass. Chromosomes are shown in order of size from left to right and top to bottom. An interactive version of this figure may be viewed at https://genome-note-higlass.tol.sanger.ac.uk/l/?d=CCsDWw10Qt-VDVvAJvS_-w.

Manual assembly curation corrected three missing joins or mis-joins and one haplotypic duplications, increasing the scaffold number by 1.92%. The final assembly has a total length of 586.80 Mb in 52 sequence scaffolds with a scaffold N50 of 20.3 Mb ( Table 2). The snail plot in Figure 2 provides a summary of the assembly statistics, while Figure 3 shows the base coverage against position per chromosome of the assembly, with the sequences coloured by phylum. The cumulative assembly plot in Figure 4 shows curves for subsets of scaffolds assigned to different phyla. Most (99.72%) of the assembly sequence was assigned to 31 chromosomal-level scaffolds, representing 30 autosomes and the Z sex chromosome. Chromosome-scale scaffolds confirmed by the Hi-C data are named in order of size ( Figure 5; Table 3). While not fully phased, the assembly deposited is of one haplotype. Contigs corresponding to the second haplotype have also been deposited. The mitochondrial genome was also assembled and can be found as a contig within the multifasta file of the genome submission.

**Table 2.** Genome assembly data for *Diarsia brunnea*, ilDiaBrun1.1.

Genome assembly		
Assembly name	ilDiaBrun1.1	
Assembly accession	GCA_949774965.1	
*Accession of alternate haplotype*	*GCA_949774955.1*	
Span (Mb)	586.80	
Number of contigs	153	
Contig N50 length (Mb)	6.7	
Number of scaffolds	52	
Scaffold N50 length (Mb)	20.3	
Longest scaffold (Mb)	36.43	
Assembly metrics *		*Benchmark*
Consensus quality (QV)	67.1	*≥ 50*
*k*-mer completeness	100.0%	*≥ 95%*
BUSCO **	C:98.8%[S:98.3%,D:0.5%], F:0.2%,M:1.0%,n:5,286	*C ≥ 95%*
Percentage of assembly mapped to chromosomes	99.72%	*≥ 95%*
Sex chromosomes	Z	*localised homologous pairs*
Organelles	Mitochondrial genome: 15.29 kb	*complete single alleles*
Genome annotation of assembly GCA_949774965.1 at Ensembl		
Number of protein-coding genes	18,730	
Number of gene transcripts	18,933	

* Assembly metric benchmarks are adapted from column VGP-2020 of “Table 1: Proposed standards and metrics for defining genome assembly quality” from Rhie *et al.* (2021). ** BUSCO scores based on the lepidoptera_odb10 BUSCO set using version 5.3.2. C = complete [S = single copy, D = duplicated], F = fragmented, M = missing, n = number of orthologues in comparison. A full set of BUSCO scores is available at https://blobtoolkit.genomehubs.org/view/ilDiaBrun1_1/dataset/ilDiaBrun1_1/busco.

**Table 3.** Chromosomal pseudomolecules in the genome assembly of *Diarsia brunnea*, ilDiaBrun1.

INSDC accession	Name	Length (Mb)	GC%
OX458997.1	1	23.35	38.0
OX458998.1	2	22.88	38.0
OX458999.1	3	22.07	38.0
OX459000.1	4	21.91	38.0
OX459001.1	5	21.8	38.0
OX459002.1	6	21.36	38.0
OX459003.1	7	21.13	37.5
OX459004.1	8	21.1	38.0
OX459005.1	9	20.98	38.0
OX459006.1	10	20.53	37.5
OX459007.1	11	20.38	38.5
OX459008.1	12	20.34	38.0
OX459009.1	13	20.04	38.0
OX459010.1	14	19.98	38.0
OX459011.1	15	19.61	38.0
OX459012.1	16	19.54	38.0
OX459013.1	17	18.83	38.5
OX459014.1	18	18.83	38.0
OX459015.1	19	18.81	38.5
OX459016.1	20	18.7	38.5
OX459017.1	21	18.19	38.5
OX459018.1	22	17.28	38.0
OX459019.1	23	15.9	38.0
OX459020.1	24	14.83	38.5
OX459021.1	25	13.74	38.0
OX459022.1	26	13.62	38.5
OX459023.1	27	10.99	39.0
OX459024.1	28	10.9	39.0
OX459025.1	29	10.83	39.5
OX459026.1	30	10.28	39.0
OX458996.1	Z	36.43	38.0
OX459027.1	MT	0.02	19.5

The estimated Quality Value (QV) of the final assembly is 67.1 with *k*-mer completeness of 100.0%, and the assembly has a BUSCO v5.3.2 completeness of 98.8% (single = 98.3%, duplicated = 0.5%), using the lepidoptera_odb10 reference set ( *n* = 5286).

Metadata for specimens, BOLD barcode results, spectra estimates, sequencing runs, contaminants and pre-curation assembly statistics are given at https://links.tol.sanger.ac.uk/species/987923.

## Genome annotation report

The *Diarsia brunnea* genome assembly (GCA_949774965.1) was annotated at the European Bioinformatics Institute (EBI) on Ensembl Rapid Release. The resulting annotation includes 18,933 transcribed mRNAs from 18,730 protein-coding genes ( Table 2; https://rapid.ensembl.org/Diarsia_brunnea_GCA_949774965.1/Info/Index). The average transcript length is 7,588.80. There are 1.01 coding transcripts per gene and 5.47 exons per transcript.

## Methods

### Sample acquisition and nucleic acid extraction

An adult *Diarsia brunnea* (specimen ID SAN00002568, ToLID ilDiaBrun1) was collected from Little Sparta, Dunsyre, Pentland Hills, Scotland, UK (latitude 55.72, longitude –3.51) on 2022-06-17 using a mercury vapour moth trap. The specimen was collected and identified by Jo Davis (Butterfly Conservation) and then preserved by flash freezing.

The specimen used for Hi-C and RNA sequencing (specimen ID SAN00002599, ToLID ilDiaBrun2) was collected from Isle of Bute, Mount Stuart, Scotland, UK (latitude 55.8, longitude –5.03) on 2022-07-02. The specimen was collected and identified by Dougie Menzies (Bute Natural History Society) and preserved on dry ice.

The workflow for high molecular weight (HMW) DNA extraction at the Wellcome Sanger Institute (WSI) Tree of Life Core Laboratory includes a sequence of core procedures: sample preparation; sample homogenisation, DNA extraction, fragmentation, and clean-up. In sample preparation, the ilDiaBrun1 sample was weighed and dissected on dry ice ( Jay *et al.*, 2023). Tissue from the thorax was homogenised using a PowerMasher II tissue disruptor ( Denton *et al.*, 2023a).

HMW DNA was extracted in the WSI Scientific Operations core using the Automated MagAttract v2 protocol ( Oatley *et al.*, 2023). The DNA was sheared into an average fragment size of 12–20 kb in a Megaruptor 3 system ( Bates *et al.*, 2023). Sheared DNA was purified by solid-phase reversible immobilisation ( Strickland *et al.*, 2023): in brief, the method employs AMPure PB beads to eliminate shorter fragments and concentrate the DNA. The concentration of the sheared and purified DNA was assessed using a Nanodrop spectrophotometer and Qubit Fluorometer using the Qubit dsDNA High Sensitivity Assay kit. Fragment size distribution was evaluated by running the sample on the FemtoPulse system.

RNA was extracted from abdomen tissue of ilDiaBrun2 in the Tree of Life Laboratory at the WSI using the RNA Extraction: Automated MagMax™ *mir*Vana protocol ( do Amaral *et al.*, 2023). The RNA concentration was assessed using a Nanodrop spectrophotometer and a Qubit Fluorometer using the Qubit RNA Broad-Range Assay kit. Analysis of the integrity of the RNA was done using the Agilent RNA 6000 Pico Kit and Eukaryotic Total RNA assay.

Protocols developed by the WSI Tree of Life laboratory are publicly available on protocols.io ( Denton *et al.*, 2023b).

### Sequencing

Pacific Biosciences HiFi circular consensus DNA sequencing libraries were constructed according to the manufacturers’ instructions. Poly(A) RNA-Seq libraries were constructed using the NEB Ultra II RNA Library Prep kit. DNA and RNA sequencing was performed by the Scientific Operations core at the WSI on Pacific Biosciences Sequel IIe (HiFi) and Illumina NovaSeq X (RNA-Seq) instruments. Hi-C data were also generated from head tissue of ilDiaBrun2 using the Arima-HiC v2 kit. The Hi-C sequencing was performed using paired-end sequencing with a read length of 150 bp on the Illumina NovaSeq 6000 instrument.

### Genome assembly, curation and evaluation

***Assembly***

The original assembly of HiFi reads was performed using Hifiasm ( Cheng *et al.*, 2021) with the --primary option. Haplotypic duplications were identified and removed with purge_dups ( Guan *et al.*, 2020). Hi-C reads were further mapped with bwa-mem2 ( Vasimuddin *et al.*, 2019) to the primary contigs, which were further scaffolded using the provided Hi-C data ( Rao *et al.*, 2014) in YaHS ( Zhou *et al.*, 2023) using the --break option. Scaffolded assemblies were evaluated using Gfastats ( Formenti *et al.*, 2022), BUSCO ( Manni *et al.*, 2021) and MERQURY.FK ( Rhie *et al.*, 2020).

The mitochondrial genome was assembled using MitoHiFi ( Uliano-Silva *et al.*, 2023), which runs MitoFinder ( Allio *et al.*, 2020) and uses these annotations to select the final mitochondrial contig and to ensure the general quality of the sequence.

***Assembly curation***

The assembly was decontaminated using the Assembly Screen for Cobionts and Contaminants (ASCC) pipeline (article in preparation). Manual curation was primarily conducted using PretextView ( Harry, 2022), with additional insights provided by JBrowse2 ( Diesh *et al.*, 2023) and HiGlass ( Kerpedjiev *et al.*, 2018). Scaffolds were visually inspected and corrected as described by Howe *et al*. (2021). Any identified contamination, missed joins, and mis-joins were corrected, and duplicate sequences were tagged and removed. The entire process is documented at https://gitlab.com/wtsi-grit/rapid-curation (article in preparation).

***Evaluation of the final assembly***

A Hi-C map for the final assembly was produced using bwa-mem2 ( Vasimuddin *et al.*, 2019) in the Cooler file format ( Abdennur & Mirny, 2020). To assess the assembly metrics, the *k*-mer completeness and QV consensus quality values were calculated in Merqury ( Rhie *et al.*, 2020). This work was done using the “sanger-tol/readmapping” ( Surana *et al.*, 2023a) and “sanger-tol/genomenote” ( Surana *et al.*, 2023b) pipelines. The genome readmapping pipelines were developed using the nf-core tooling ( Ewels *et al.*, 2020), use MultiQC ( Ewels *et al.*, 2016), and make extensive use of the Conda package manager, the Bioconda initiative ( Grüning *et al.*, 2018), the Biocontainers infrastructure ( da Veiga Leprevost *et al.*, 2017), and the Docker ( Merkel, 2014) and Singularity ( Kurtzer *et al.*, 2017) containerisation solutions. The genome was also analysed within the BlobToolKit environment ( Challis *et al*., 2020) and BUSCO scores ( Manni *et al*., 2021; Simão *et al*., 2015) were calculated.

Table 4 contains a list of relevant software tool versions and sources.

**Table 4.** Software tools: versions and sources.

Software tool	Version	Source
BlobToolKit	4.2.1	https://github.com/blobtoolkit/blobtoolkit
BUSCO	5.3.2	https://gitlab.com/ezlab/busco
bwa-mem2	2.2.1	https://github.com/bwa-mem2/bwa-mem2
Gfastats	1.3.6	https://github.com/vgl-hub/gfastats
Hifiasm	0.16.1-r375	https://github.com/chhylp123/hifiasm
HiGlass	1.11.6	https://github.com/higlass/higlass
Merqury.FK	d00d98157618f4e8d1a9 190026b19b471055b22e	https://github.com/thegenemyers/MERQURY.FK
MitoHiFi	2	https://github.com/marcelauliano/MitoHiFi
PretextView	0.2	https://github.com/wtsi-hpag/PretextView
purge_dups	1.2.3	https://github.com/dfguan/purge_dups
sanger-tol/genomenote	v1.0	https://github.com/sanger-tol/genomenote
sanger-tol/readmapping	1.1.0	https://github.com/sanger-tol/readmapping/tree/1.1.0
YaHS	yahs-1.1.91eebc2	https://github.com/c-zhou/yahs

### Genome annotation

The BRAKER2 pipeline ( Brůna *et al.*, 2021) was used in the default protein mode to generate annotation for the *Diarsia brunnea* assembly (GCA_949774965.1) in Ensembl Rapid Release at the EBI.

### Wellcome Sanger Institute – Legal and Governance

The materials that have contributed to this genome note have been supplied by a Darwin Tree of Life Partner. The submission of materials by a Darwin Tree of Life Partner is subject to the **‘Darwin Tree of Life Project Sampling Code of Practice’**, which can be found in full on the Darwin Tree of Life website here. By agreeing with and signing up to the Sampling Code of Practice, the Darwin Tree of Life Partner agrees they will meet the legal and ethical requirements and standards set out within this document in respect of all samples acquired for, and supplied to, the Darwin Tree of Life Project. 

Further, the Wellcome Sanger Institute employs a process whereby due diligence is carried out proportionate to the nature of the materials themselves, and the circumstances under which they have been/are to be collected and provided for use. The purpose of this is to address and mitigate any potential legal and/or ethical implications of receipt and use of the materials as part of the research project, and to ensure that in doing so we align with best practice wherever possible. The overarching areas of consideration are:

• Ethical review of provenance and sourcing of the material

• Legality of collection, transfer and use (national and international) 

Each transfer of samples is further undertaken according to a Research Collaboration Agreement or Material Transfer Agreement entered into by the Darwin Tree of Life Partner, Genome Research Limited (operating as the Wellcome Sanger Institute), and in some circumstances other Darwin Tree of Life collaborators.

## Data Availability

European Nucleotide Archive: *Diarsia brunnea* (purple clay). Accession number PRJEB60714; https://identifiers.org/ena.embl/PRJEB60714 ( Wellcome Sanger Institute, 2023). The genome sequence is released openly for reuse. The *Diarsia brunnea* genome sequencing initiative is part of the Darwin Tree of Life (DToL) project. All raw sequence data and the assembly have been deposited in INSDC databases. Raw data and assembly accession identifiers are reported in Table 1 and Table 2.

## Author information

Members of the Wellcome Sanger Institute Tree of Life Management, Samples and Laboratory team are listed here: https://doi.org/10.5281/zenodo.12162482.

Members of Wellcome Sanger Institute Scientific Operations: Sequencing Operations are listed here: https://doi.org/10.5281/zenodo.12165051.

Members of the Wellcome Sanger Institute Tree of Life Core Informatics team are listed here: https://doi.org/10.5281/zenodo.12160324.

Members of the Tree of Life Core Informatics collective are listed here: https://doi.org/10.5281/zenodo.12205391.

Members of the Darwin Tree of Life Consortium are listed here: https://doi.org/10.5281/zenodo.4783558.
