# Supplementary material for: The genome sequence of the Purple Clay moth, Diarsia brunnea (Denis & Schiffermüller) 1775
Source: Wellcome Open Res. 2024 Aug 14;9:471. [Version 1] doi: 10.12688/wellcomeopenres.22868.1 (PMC11413557; doi:10.12688/wellcomeopenres.22868.1)
Supplement: Peer review history

# Reviewer report: Reviewer response for version 1

**DOI:** 10.21956/wellcomeopenres.25181.r97600

**Published:** 2024-09-19

**Authors:** Inusa Jacob Ajene, Fathiya Khamis

**Affiliations:** 1 International Centre for Insect Physiology and Ecology, Nairobi, Nairobi County, Kenya

The data note titled “The genome sequence of the Purple Clay moth, Diarsia brunnea (Denis & Schiffermüller) 1775” presents the genome assembly of the Purple Clay moth. This moth has a wide distribution range across Europe and Asia. This is a good study with impact as a potential resource for future genomic studies, including population genetics and phylogeography of the moth. The methodology describing the genome assembly and annotation is sound, and the results present a comprehensive view of the study genome. The background of the insect is interesting; however, a minor addition would be a few lines to justify the study of the nuclear genome of the moth.

Are sufficient details of methods and materials provided to allow replication by others?

Yes

Is the rationale for creating the dataset(s) clearly described?

Partly

Are the datasets clearly presented in a useable and accessible format?

Yes

Are the protocols appropriate and is the work technically sound?

Yes

Reviewer Expertise:

Molecular biology and Bioinformatics

We confirm that we have read this submission and believe that we have an appropriate level of expertise to confirm that it is of an acceptable scientific standard.

# Reviewer report: Reviewer response for version 1

**DOI:** 10.21956/wellcomeopenres.25181.r95727

**Published:** 2024-09-06

**Authors:** Andrew A Walker

**Affiliations:** 1 University of Queensland, Queensland, Australia

This report is of a high-quality nuclear genome sequencing and assembly of the lepidopteran Diarsia brunnea. It has been clearly prepared and I don't have specific suggestions to change it.

 I am not super familiar with what Wellcome Open Research requires in an article. This indexing just describes the sequencing and assembly of the genome, and does not analyse it further in a way relevant to lepidopteran biology. However I get the vibe that it is part of a larger project to enlarge genomic resources across the tree of life in general. Perhaps this could be clarified in the Background with a very minor change.

Are sufficient details of methods and materials provided to allow replication by others?

Yes

Is the rationale for creating the dataset(s) clearly described?

Partly

Are the datasets clearly presented in a useable and accessible format?

Yes

Are the protocols appropriate and is the work technically sound?

Yes

Reviewer Expertise:

Omics technologies

I confirm that I have read this submission and believe that I have an appropriate level of expertise to confirm that it is of an acceptable scientific standard.
